# Non-surgical endodontics in retreatment of periapical lesions – two representative case reports

**DOI:** 10.4317/jced.50765

**Published:** 2012-07-01

**Authors:** Varun Kapoor, Samrity Paul

**Affiliations:** 1 B.D.S., M.D.S. Conservative Dentistry and Endodontics. Senior Lecturer, Swami Devi Dyal Hospital and Dental College, Golpura, Panchkula.; 2 B.D.S., M.D.S. Oral and Maxillofacial Surgery, Senior Lecturer, Swami Devi Dyal Hospital and Dental College, Golpura, Panchkula.

## Abstract

This article reports non-surgical endodontic retreatment of two patients with persistent or recurrent periapical lesions, who had previously undergone surgical and non-surgical endodontic therapy respectively. It further discusses and reviews the relevance of classification of periapical lesions, the explanation behind healing of periapical lesions by endodontic therapy alone, causes of persistence of periapical lesions, choice of treatment modalities (whether surgical or non – surgical) and materials such as intracanal medicaments and irrigants for optimal healing.

** Key words:**Non-surgical, retreatment, periapical, calcium hydroxide, chlorhexidine irrigation.

## Introduction

The classification of periapical lesions into abscesses, granulomata and cysts is common knowledge. Many authors have described criteria to differentiate granulomas from cysts. Characteristics suggestive of cysts include size (60-67% incidence of cysts in periapical lesions greater than 10mm (1,2). 92% incidence of cysts in periapical lesions greater than 200mm (2,3)), involvement of multiple teeth with necrotic pulp, straw colored aspirate or drainage (4), cholesterol crystals (4,5) etc.

However, the relevance of such classification in endodontic practice is considerably controversial. Upto 85% treatment success has been reported for periapical lesions after endodontic therapy alone (6,7,8). which implies that most periapical lesions including cysts respond to endodontic therapy alone, as also discussed by Nair (1999) (9). This is explained by the effect of biomechanical preparation on intracanal microbiota, (10,11) enzy-matic mechanisms, (12) immunological mechanisms involving neutralization of antigenic toxins, (13,14,15) and breakdown of epithelial lining with involvement of macrophages, non killer T lymphocytes and Langerhans cells (10,16). Pocket cysts heal due to removal of intracanal irritants, as they have an open lumen to the root canal system (10). Since it is clinic-radio graphically not always possible to distinguish cysts from granulomas, or true and pocket cyst, conservative endodontics should be the first line of treatment.

Retreatment of periapical lesions requires variations in the same thought process. Options for re-treatment, too, are non-surgical endodontic retreatment or surgical endodontics. Especially in cases of improper or defective filling of the canal system, the root canal and periapex should respond to orthograde treatment. The choice of treatment approach should be based upon the patient’s clinical situation and preference, operator’s experience and skill, the risk of complications, and the technical feasibility and cost (17).

In this vein, these representative cases of patients undergoing non-surgical endodontic retreatment have been reported and non surgical endodontic retreatment as a modality has been discussed.

## Case Report 1

This 30 year old gentleman presented with swelling in his anterior palate since 2 months. He gave history of trauma to the upper anterior teeth in a fall 4 years back. He had developed palatal swelling and discoloration of an anterior tooth 1 year back and undergone endodontic therapy, followed by surgical intervention and full coverage prosthesis. The patient remained asymptomatic till reappearance of the palatal swelling, which precipitated his visit to the hospital.

**Figure 1:**
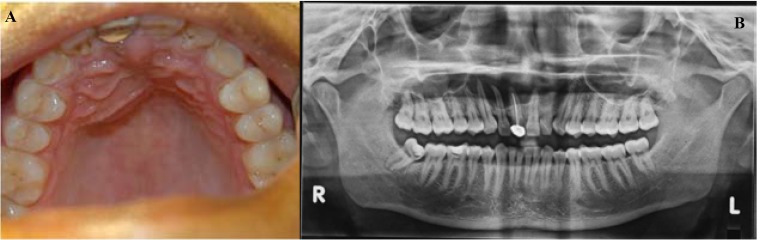
A.-Palatal swelling. B.-Orthopantomogram.

Intraorally, a firm swelling of the right palate, 1 cm in diameter, was obvious. The right maxillary central incisor, which was tender, had been restored with a full-coverage porcelain fused to metal crown. The adjacent incisors (maxillary right lateral and left central) were also tender, and non-responsive to thermal and electric pulp testing (Fig. 1).

Radiographs (periapical, maxillary occlusal and Orthopantomogram) revealed a unilocular periapical radiolu-cency with smooth, sclerotic borders, 1.5 cm in widest dimension, and involving the periapices of incisors. Glaringly obvious was the unfortunate obturation of the incisor, which was a good 4-5 mm short of length and grossly underfilled.

The patient was offered three treatment choices – non surgical endodontic therapy, periapical surgery, or extraction and prosthetic replacement. The gentleman was averse to surgery and opted for non – surgical treatment. He was further keen to preserve the existing crown, as he was satisfied with its function.

The root canal system of the right central incisor was accessed through the coronal prosthesis, and the old gutta percha removed (utilizing Gates Glidden drills, hand instrumentation with Hedstrom files and Endosolv gutta-percha solvent). The canal yielded purulent fluid exudate. The adjacent incisors, on access opening, also exhibited purulent drainage. After about an hour, frank drainage had subsided, so the access preparations were sealed with a temporary filling material (Coltsol, Coltene, Altstatten, Switzerland).

The next day, the swelling had, miraculously, disappeared completely. Working lengths were determined for all three teeth electronically and radiographically. The canals were prepared by a step-back technique with K-type files (Dentsply Maillefer, Ballaigues, Switzerland). The irrigants (2.5% sodium hypochlorite and 0.2% chlor-hexidine) were delivered carefully and passively. RC Prep (Premier Dental Company, Plymouth Meeting, PA, USA) was used to remove the smear layer prior to a final flush with 0.2% chlorhexidine.

After chemomechanical preparation and drying of root canal system, a slurry of calcium hydroxide powder and 2% chlorhexidine (gel) was introduced to sterilize the canal, and retained for 2 weeks. Finally, the root canal systems of the incisors were obturated with gutta-percha and AH plus sealer (De Trey, Konstanz, Germany) using a lateral compaction technique.

**Figure 2:**
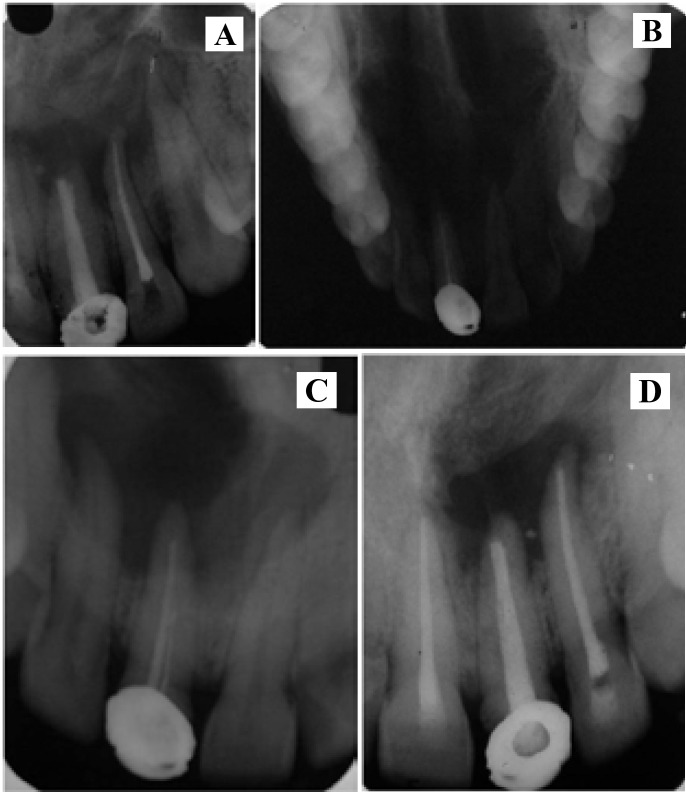
A.- Maxillary occlusal view. B. - Pre-operative Intraoral periapical radiograph. C. - months post operative radiograph. D. - 6 months post operative radiograph.

The patient’s symptomatic relief was immediate. Six months post operatively, periapical radiograph showed significant reduction in lesion size and appearance of new trabecular pattern in the region (Fig. 2).

## Case Report 2

A 23-year-old girl was referred for the treatment of persistent mild pain and intermittent swelling in the mandibular anterior tooth region. The patient had met with a road accident when she was 7 years old and had under root canal treatment of her mandibular incisors. The patient was unable to produce previous treatment records or recall all treatment details.

Clinical examination of soft tissues showed no discoloration, scarring or fistulae. All four mandibular incisors were malaligned; both mandibular central and lateral incisors were slightly sensitive to percussion and palpation, and had grade I mobility.

Periapical radiographs revealed large periapical lesions with ill defined borders and incompletely formed root apices of all four mandibular incisors. The root fills in these four teeth were remarkably deficient in all three dimensions, and ridiculously short of working length in three teeth.

**Figure 3:**
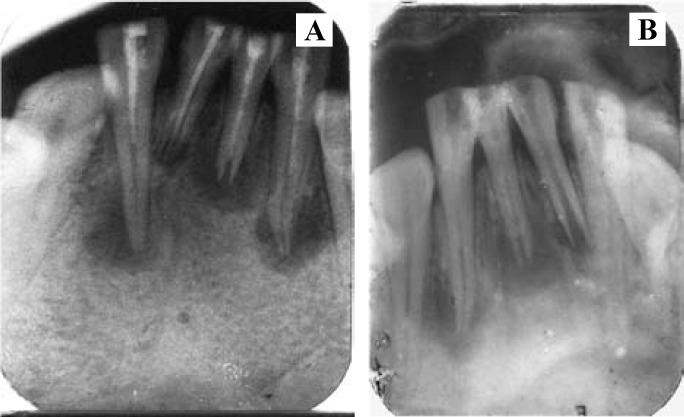
A. - Pre-operative Intraoral periapical radiograph. B. - After removal of old gutta-percha and sealer.

Non-surgical endodontic therapy was planned for all mandibular incisors. A purulent fluid (blood tinged) drained through the canals on access opening. When the drainage ceased, old gutta-percha was removed, working lengths determined and the canals biomechanically prepared by a method identical to the first case report (Fig. 3).

**Figure 4:**
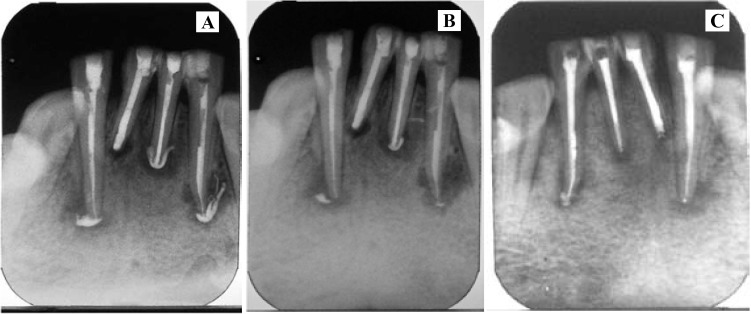
A. Three months after Calcium hydroxide paste insertion. B. Six months after Calcium hydroxide paste insertion. C. - Obturation at nine months.

After chemomechanical preparation of root canal system, non-setting calcium hydroxide paste (Metapex, Meta Biomed Co.Ltd., Chungcheongbuk-do, South Korea) was dispensed into the canals. Some of the paste extruded unavoidably through the open apex into the peri-apex. Access cavity was sealed with Coltsol. After the second change of dressing, there was no intracanal exudate, the patient was asymptomatic, and the periapical radiolu-cency reduced progressively. The intracanal dressings were to be changed monthly, but poor patient compliance resulted in only three dressing changes in 6 months. The extruded metapex had fortunately undergone resorption by this time. Apexification was also radiologically substantiable. The tooth was obturated by lateral compaction after 9 months with gutta-percha and AH plus sealer, when the periapical healing was thought to be adequate. The patient is being followed up ever since, to observe further periapical healing (Fig. 4).

## Discussion

Many factors may result in persistence of periapical radiolucencies, such as (i) intraradicular infection (due to coronal / apical leakage, inadequate root fillings, incomplete biomechanical preparation, poor root canal treatment), (ii) extraradicular infections (from canal system or in the periapex itself), (iii) foreign body reaction (to cholesterol crystals, talc contaminated guttapercha, cellulose of paper points, sealing or obturating material etc.), (iv) true cysts or (v) fibrous scar tissue (18).

A comparison of the two retreatment techniques (surgical and non-surgical) over time by Torabinejad et al (2009) (20) showed a significantly higher success rate for endodontic surgery (77.8%) at 2-4 years in comparison to non surgical endodontics (71.9%), and an equally significant role reversal at 4-6 years, with non-surgical retreatment showing a much higher success rate of 83% compared to surgical results (71.8%). Where non surgical endodontics showed a rise in weighted success with time, endodontic surgery showed an obvious decline (to 62.9% at 6 years and above).

History of endodontic periapical surgery is a further complicating factor in retreatment. Bacteria may be harbo-red in pre-existing apical end resorption lacunae (19). Apicocoectomy results in a root end similar to an open apex or Blunderbuss canal. Further problems may arise from increased permeability of dentinal tubules, influence of smear layer on the cut surface, or incorrect placement of retrograde material (19). All these factors predispose the periapex to residual infection.

Calcium hydroxide is indisputably the most appropriate intracanal medicament for teeth with periapical lesions, as it removes micro-organisms and promotes repair by controlling the inflammatory action (by hygroscopic action, calcium proteinate bridge formation, phosphidase inhibition), neutralizing osteoclasts acid products (acid hydrolases and lactic acid), inducing cellular differentiation (alkaline phosphatase activation and calcium de-pendent ATPases) and neutralization of exotoxins (10).

Amongst Irrigating solutions, both sodium hypochlorite and calcium hydroxide are ineffective against E. fecalis and C. albicans, limiting their usage in periapical lesions (18). Chlorhexidine has been found an efficacious irrigating agent against these microbes, both in 0.5 and 2% formulations, as solution and gel. Moreover, the gel form compensates for its inability to dissolve pulpal tissue and smear layer by promoting a better mechanical cleansing of the canal system (21). Chlorhexidine can be used as a disinfecting last rinse after biomechanical preparation with sodium hypochlorite (22). Iodine potassium iodide and MTAD (Mixture of Tetracycline isomer, citric acid and detergent) are also being reinvestigated in recent years owing to their effect against E. fecalis.

The prognosis of retreatment is not 100% either. It worsens with root canal morphology altered by previous treatment, greater size of lesion, preoperative perforation, over instrumentation, over filling, poor biomechanical preparation, root fill, coronal or apical seal etc.

These two cases illustrate the scope of non-surgical endodontics. A longer follow-up is advisable to observe progressing healing or recurrence. Neoplastic or carcinomatous transformation of periapical cysts has extremely few antecedents, (23,24) but is also something to be borne in mind while advising follow-up.

## References

[1] Lalonde ER, Luebke RG (1968). The frequency and distribution of periapical cysts and granulomas. An evaluation of 800 specimens. Oral Surg Oral Med Oral Pathol.

[2] Morse DR, Patnik IW, Schacterlie GR (1973). Electrophoretic differentiation of radicular cysts and granulomas. Oral Surgery, Oral Medicine and Oral Pathology.

[3] Zain RB, Roswati N, Ismail K (1989). Radiographic features of periapical cysts and granulomas. Singapore Dental Journal.

[4] Shear M, Speight P (2007). Cysts of the Oral and Maxillofacial Regions.

[5] Eversole RL (2002). Clinical Outline of Oral Pathology: Diagnosis and Treatment.

[6] Bhaskar SN (1966). Periapical lesions-types, incidence, and clinical features. Oral Surg Oral Med Oral Pathol.

[7] Sjogren U, Hagglund B, Sundqvist G, Wing K (1990). Factors affecting the long-term results of endodontic treatment. J Endod.

[8] Caliskan MK, Sen Bz (1996). Endodontic treatment of teeth with apical periodontitis using calcium hydroxide: a long-term study. Endodontics and Dental Traumatology.

[9] Nair PN, Sjogren U, Figdor D, Sundqvist G (1999). Persistent periapical radiolucencies of root-filled human teeth, failed endodontic treatments, and periapical scars. Oral Surg Oral Med Oral Pathol Oral Radiol Endod.

[10] Soares J, Santos S, Silveira F, Nunes E (2006). Nonsurgical treatment of extensive cyst-like periapical lesion of endodontic origin. Int Endod J.

[11] Bhaskar SN (1972). Nonsurgical resolution of radiculer cysts. Oral Surgery, Oral Medicine and Oral Pathology.

[12] Catanzaro-Guimara SA, Alle N (1973). Observations on the structure and pathogenesis of apical periodontal cyst. Estomatol Cult.

[13] Tronstad L, Andreasen JO, Hasselgren G, Kristerson L, Riis I (1981). pH changes in dental tissues after root canal filling with calcium hydroxide. Journal of Endodontics.

[14] Seux D, Couble ML, Hartmann DJ, Gauthier JP, Magloire H (1991). Odontoblast-like cytodifferentiation of human dental pulp cells in vitro in the presence of calcium hydroxide-containing cement. Archives of Oral Biology.

[15] Safavi KE, Nichols FC (1993). Effect of calcium hydroxide on bacterial lipopolysaccharide. Journal of Endodontics.

[16] Kettering JD, Torabinejad M (1993). Presence of natural killer cells in human chronic periapical lesions. International Endodontic Journal.

[17] Del Fabbro M, Taschieri S, Testori T, Francetti L, Weinstein RL (2007). Surgical versus non-surgical endodontic re-treatment for periradicular lesions. Aust Dent J.

[18] Yan MT (2006). The management of periapical lesions in endodontically treated teeth. Aust Endod J.

[19] Fava LRG (2001). Calcium hydroxide in endodontic retreatment after two nonsurgical and two surgical failures: report of a case. Int Endod J.

[20] Torabinejad M, Corr R, Handysides R, Shabahang S (2009). Outcomes of Nonsurgical Retreatment and Endodontic Surgery: A Systematic Review. J Endod.

[21] Ferraz CC, Figueiredo de Almeida Gomes BP, Zaia AA, Teixeira FB, de Souza-Filho FJ (2001). In vitro assessment of the antimicrobial action and the mechanical ability of chlorhexidine gel as an endodontic irrigant. J Endod.

[22] Zamany A, Safavi K, Spangberg LS (2003). The effect of chlorhexidine as an endodontic disinfectant. Oral Surg Oral Med Oral Pathol Oral Radiol Endod.

[23] Van der Waal I, Rauhamaa R (1985). van der Kwast W.A.M. and Snow G.B. Squamous cell carcinoma arising in the lining of odontogenic cysts. Report of 5 cases. International Journal of Oral Surgery.

[24] Swinson BD, Jerjes W, Thomas GJ (2005). Squamous cell carcinoma arising in a residual odontogenic cyst: case report. Journal of Oral and Maxillofacial Surgery.

